# Flow-based regenerable chemiluminescence receptor assay for the detection of tetracyclines

**DOI:** 10.1007/s00216-019-02368-y

**Published:** 2020-01-16

**Authors:** Verena K. Meyer, Claire V. Chatelle, Wilfried Weber, Reinhard Niessner, Michael Seidel

**Affiliations:** 1 Institute of Hydrochemistry, Chair of Analytical Chemistry and Water Chemistry, Technical University of Munich, Marchioninistraße 17, 81377 Munich, Germany; 2 Faculty of Biology and Signalling Research Centres BIOSS and CIBSS, University of Freiburg, Schänzlestraße 18, 79104 Freiburg, Germany

**Keywords:** Receptor assay, Antibiotics, Tetracyclines, Chemiluminescence microarray, Regenerable biosensor

## Abstract

For the first time, a flow-based regenerable chemiluminescence receptor assay is established that is eminently suited as screening method for the detection of widely used tetracyclines (TCs) in environmental and food samples. The complex functionality and high reactivity of TCs complicate the creation of immunogens which is currently the bottleneck for developing sensitive immunoassays. In this case, competitive bioreceptor assays for the analysis of small organic molecules are preferable and, moreover, flow-based regenerable bioassays are optimally suited for automated analysis applications. Therefore, the solution for rapid and sensitive analysis of TCs is the regenerable CL receptor assay with a covalently immobilized DNA oligonucleotide containing the specific operator sequence *tetO* to which the repressor protein TetR binds only in the absence of TCs. The TC measurements are performed on the CL microarray analysis platform MCR 3 within 30 min per sample. The LoD in spiked tap water was determined to be 0.1 μg L^−1^, and for 1 μg L^−1^ TET, recoveries of 77% ± 16% were obtained. Due to the stability of the immobilized DNA oligonucleotide and the resulting regenerability of the assay for various measurements, the new method is highly cost- and resource-efficient and ideally suited for the monitoring of environmental samples in the field.

**Figure:**
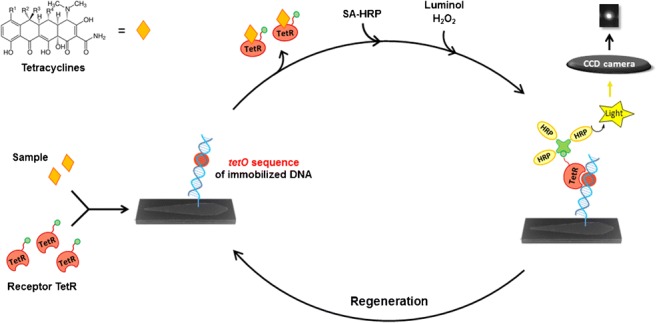
Graphical abstract

Graphical abstract

## Introduction

TCs are natural or semisynthetic antibiotics that act against a wide range of gram-positive and gram-negative bacteria and other microorganisms [1, 2]. They are the most common veterinary antibiotics worldwide [3], such as in 2016, 5.9 kt TCs were sold in the USA and 2.5 kt in Europe (42% and 32% of the veterinary antibiotic sales amount, respectively) [4, 5]. Various livestock breeds as poultry, cattle, swine, and sheep as well as aquacultures are treated with TCs to prevent or cure microbial infections and, in many countries, they are also used as growth promotors [2, 6, 7]. To attend whole herds, the drug is directly added to feed or water or even spread by aerosols [2]. In human medicine, TCs are mainly applied to treat atypical pneumonia, chlamydial infections, acute Q fever, and acne vulgaris [1]. Due to the low costs, TCs are particularly relevant for economically weak regions of the world [2]. Over the past decades, the number of TC-resistant bacterial strains drastically increased, so the ubiquitous presence of TCs and TC-resistant bacteria is an issue of public health [2, 8].

The TCs administered in animal husbandry are released into the environment via excretion and using the excrements as manure on the fields. So TCs have already been found in various environmental samples. Due to their chelating ability, TCs are mainly accumulated in soil fertilized with manure or in sediment but have also been detected in surface waters [9, 10]. Remarkably high maximum concentrations were found in arable soil, e.g., in northern China (OTC 2700 μg kg^−1^) [11], northern Germany (TET 307 μg kg^−1^) [12], and South Korea (TET 178 μg kg^−1^) [13]. In surface waters, high concentrations were detected in South Korea (TET 255 μg L^−1^) [13], China (OTC 73 μg L^−1^) [14], Japan (OTC 68 μg L^−1^) [15], and USA (OTC 1.3 μg L^−1^) [16]. For detailed reviews of multiple studies, it is referred to [3, 9].

The most common confirmatory method for the analysis of TCs is HPLC-MS [8, 17]. Disadvantages are high costs of apparatus and supplies, complex sample preparation, and the need for skilled personnel [17, 18]. For an extensive monitoring of TC residues in environmental and food samples, cost-efficient and easy-to-handle high-throughput screening methods with low detection limits are required [17, 19]. Microbiological inhibition tests are very common screening methods for the detection of various antimicrobial substances [17]. The growth of test microorganisms is inhibited when antibiotics are present [20]. Some advantages are simplicity and low costs as well as the possibility to detect any substance with antimicrobial activity [17, 18]. Therefore, several commercial test kits are available as screening methods for food analyses [17]. Though, these inhibition tests are with several hours very time-consuming and non-selective [17, 18]. An advanced method in biosensor format detected the inhibition of bacterial growth based on decreasing CO_2_ production at a concentration level of 25 μg L^−1^ TCs in milk within 120 min [21]. Immunoassays are, in general, widely established in routine analysis due to their high sensitivity and selectivity combined with cost efficiency and simplicity [17, 18]. A selective antibody reacts with an antigen which is normally a characteristic section of a substance or a class of substances [17, 19]. Prior to this work, a flow-based microarray with regenerable indirect competitive CL immunoassays for the simultaneous detection of 13 antibiotics has successfully been established [22]. For the purpose of a competitive TC assay, however, it was not possible to stably immobilize a TC derivative that would have been regenerable over several measurements. Due to the complex functionality and the high reactivity of TCs, coupling a TC derivative to a carrier protein for antibody production is also quite difficult, so there are only few immunoassay methods reported for the detection of TCs [23]. Instead of antibodies, in vitro produced biomimetic receptors as MIPs and aptamers are promising tools for biosensors [19].

A wide range of antibiotic analysis methods using proteins as bioreceptors is already established. These naturally occurring proteins are selective for a certain class of substances which means that they do not distinguish between different derivatives of e.g. TCs but can be used for comprising detection of the respective antibiotic class [18]. For analytic application, either whole cells containing the receptor protein are directly used or the protein is isolated. Furthermore, the receptor protein can easily be expressed recombinantly in bacteria which is a clear advantage compared to antibodies [18]. Suitable proteins are either the target protein of the antibiotic activity in the bacterial cell or a protein that is part of the bacterial resistance mechanism of resistant bacteria. For TC detection, the repressor protein TetR that is found in TC-resistant bacteria can be used [24]. TetR binds specifically to a DNA sequence (*tetO*) and therefore inhibits the expression of the TC efflux protein TetA. Binding of TC to TetR leads to a conformational change of the receptor protein so that it releases the DNA and the efflux protein TetA is produced [25]. TetR has already been established in some analytical applications. Commercially available are e.g. SNAP (enzyme-labeling receptor assay), Charm II (radio receptor assay), and Tetrasensor (colloidal gold receptor assay) [18].

Receptor-based assays for TC detection are summarized in Table 1. First, there are several static assays (a)—(e). A whole-cell biosensor based on the TC-resistant *Escherichia coli* strain TetLux was developed for the detection of TCs in MTPs (a). The cells contained a plasmid featuring a bacterial luciferase operon that was regulated together with *tetO*. Therefore, binding of TC to TetR led to the expression of the luciferase and the bioluminescence could be detected [26]. In a competitive MTP receptor assay (b), HRP-conjugated TET was used to compete with TCs in the sample for immobilized TetR [27]. Competitive assays with immobilized *tetO* were also established. A dipstick test on membrane strips (c) was based on His_6_-tagged TetR that was released from *tetO* in presence of TCs. The remaining TetR was marked with anti-His_5_ antibody and HRP-labeled anti-IgG antibody and the detection was carried out by means of a color reaction [28]. Using *tetO* immobilized in MTPs, some ELISA-type assays were performed, e.g., with His_6_-tagged TetR, anti-His_5_ antibody, HRP-labeled anti-mouse antibody, and color reaction [24] or luciferase-tagged TetR and luminescence [29]. In general, these static assays are quite easy to perform. However, they are time-consuming and there is no possibility for online integration, assay regeneration or multiplexing for the simultaneous detection of several analytes.

**Table 1:** Bioassays for the detection of TCs using TetR as receptor protein

Assay principle	Detection method	Matrices	LoD/μg/L	Analysis time	Regenerable sensor?	Ref
*Static assays*						
(a) Whole-cell biosensor in MTP	Bioluminescence	Poultry meat	25 μg/kg	4 h	No	[26]
(b) TetR immobilized in MTP, TET-HRP as competitor	CL	Milk	8 ng/L	2 h (+ coating)	No	[27]
(c) *tetO* immobilized on membrane strips	Color reaction	Milk, meat, serum	5–10	1 h (+ dipstick production)	No	[28]
(d) *tetO* immobilized on MTP	Color reaction	Milk, bovine serum	1.9	Several hours (+ coating)	No	[24]
(e) *tetO* immobilized on MTP	Bioluminescence	Tris buffer	0.05	2 h (+ coating)	No	[29]
*Flow-based assays*						
(f) *tetO* immobilized on chip	Electric current	Human plasma	6.3	15 min	No	[30]
(g) DOX immobilized on chip as competitor	SPR	HBS-P buffer	–	ca. 15 min	Poorly	[31]
(h) *tetO* immobilized on chip	SPR	HBS-P buffer Milk (needs pretreatment)	1 15	ca. 10 min	Yes	[32]
(i) *tetO* covalently immobilized on chip	CL	Tap water	0.1	30 min	Yes	New

Furthermore, some flow-based methods (f)—(h) have been reported. A competitive assay with *tetO* immobilized on an electrochemical microfluidic polymer chip (f) was established. The sample was mixed with biotinylated TetR and avidin-glucose oxidase and flushed over the chip. The electrochemical signal of glucose oxidation was detected. This method can be multiplexed on a small scale as the microfluidic platform can process up to eight assays simultaneously. However, the biosensor is designed for single use and cannot be regenerated [30]. Assays with SPR detection, e.g., on Biacore platform, are basically regenerable. However, for an assay with immobilized DOX as competitor (g) it turned out that the signal intensity continued to decrease significantly with each regeneration step. The sensitivity of this method was not directly stated, but it seemed to be in the range of 20–100 μg L^−1^ [31]. The only regenerable assay was found to be the SPR (Biacore) assay with *tetO* immobilized via biotin tag on a streptavidin surface (h) [32]. The disadvantages of this method are the need for sample pretreatment due to matrix sensitivity and that a simultaneous processing of different assays on the same chip is not possible.

**Fig. 1:**
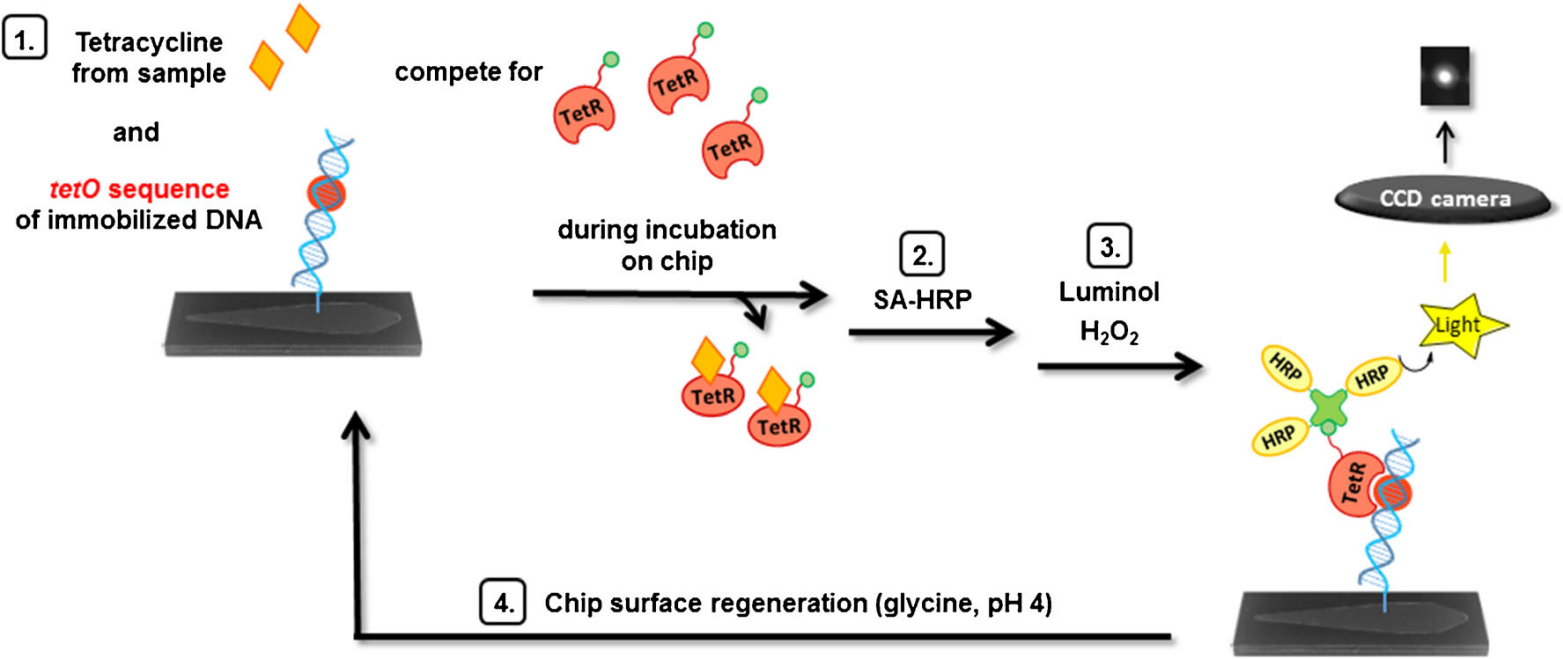
Scheme of the flow-based regenerable competitive CL assay for the detection of TCs with TetR as receptor and the immobilized DNA oligonucleotide containing the specific operator sequence *tetO* as competitor

For a fully automated on-site monitoring of several contaminants in environmental or sewage water samples, however, both multiplexing and reusability of the sensor are absolutely required. In our work, as described in Fig. 1, a double-stranded DNA oligonucleotide including the specific operator sequence *tetO* is covalently immobilized by an amino-C12 tag to a functionalized surface (a silanized and Jeffamine®-coated glass slide activated with poly(ethylene glycole) diglycidyl ether) [33]. On the microfluidic chip formed from this glass slide, a mixture of biotin-labeled TetR solution and the sample is incubated so that TC from the sample can trigger a conformational change in TetR to dissociate from immobilized *tetO*. The amount of TetR that is bound to immobilized *tetO* is marked with HRP-labeled streptavidin and the detection is achieved via HRP-catalyzed CL due to its generally high sensitivity that has already been shown for immunoassays [34, 35]. The described method presented in this work (i) is highly sensitive, robust against surface water, regenerable for at least 9 measurements, and it can easily be combined in future with various other receptor or immunoassays in a multiplex chip format.

## Materials and methods

Unless otherwise stated, all standard chemicals and materials were purchased from Sigma-Aldrich (Taufkirchen, Germany) or Carl Roth (Karlsruhe, Germany).

### Repressor protein production and purification

The repressor protein TetR was produced with a C-terminal Avitag for in vivo site specific biotinylation by the enzyme BirA, followed by a His_6_ tag for purification as described elsewhere [30]. In brief, *E. coli* BL21* (DE3) were co-transformed with plasmids pCVC008 and pBirA for co-expression of biotinylated TetR protein and BirA enzyme, respectively. Cells were grown in LB medium supplemented with ampicillin (100 μg mL^−1^) and chloramphenicol (34 μg mL^−1^) at 37 °C until OD_600_ = 0.6. The culture medium was supplemented with 50 μM biotin and protein production was induced with 1 mM β-D-1-thiogalactopyranoside overnight at 20 °C. Cells were harvested by centrifugation (6000×*g*, 10 min at room temperature), resuspended in lysis buffer (35 mL per liter initial culture volume, 50 mM NaH_2_PO_4_, 300 mM NaCl, 10 mM imidazole, pH 8.0), freeze-thawed and lysed by sonication (Bandelin, Berlin, Germany; 60%, pulse 0.5 s every second for 10 min). Subsequently, cell debris were eliminated by centrifugation (30,000×*g*, 30 min at 4 °C). Proteins were purified from the supernatant on a gravity flow Ni^2+^-NTA-agarose Superflow column (Qiagen, Hilden, Germany) following manufacturer instructions. Protein concentration was determined by the Bradford method (Bio-Rad, Hercules, USA) using BSA as standard. Proteins were diluted to 1 mg mL^−1^ in elution buffer (50 mM NaH_2_PO_4_, 300 mM NaCl, 250 mM imidazole, pH 8.0) containing 10% (w/v) sucrose, lyophilized and stored at − 80 °C. For each experiment, the lyophilized proteins were reconstituted in H_2_O.

The biotinylation motif was fused to the C-terminus of the TetR protein as this site was previously shown to support different fusion partners while not influencing DNA-binding or responsiveness to tetracycline [36]. Stability of DNA-immobilized biotinylated TetR was demonstrated for at least 3 months at 4 °C [30].

### Chip surface chemistry

The assay was performed on glass slides that were functionalized as described elsewhere [33]. In summary, microscopic glass slides were extensively cleaned and acidly activated for the following silanization with (3-glycidyloxypropyl)trimethoxysilane. The silanized carriers were then coated with Jeffamine® ED-2003, supplied as a sample by Huntsman (Everberg, Belgium), and the terminal amino groups reacted with the epoxy groups of poly(ethylene glycole) diglycidyl ether to form a robust PEG layer with epoxy groups as active binding sites for the immobilization of the oligonucleotide *tetO*.

### Annealing of the double-stranded oligonucleotide *tetO*

The double-stranded DNA operator *tetO* that can bind the repressor protein TetR was generated by mixing the oligonucleotides amino-C12-GCACTCCCTATCAGTGATAGAGAAACG and CGTTTCTCTATCACTGATAGGGAGTGC (both produced by Eurofins Genomics, Ebersberg, Germany) in equimolar amounts (100 μM/200 μM/400 μM) in PCR-grade ultrapure water. The mixtures were incubated at 95 °C for 5 min, followed by controlled cooling (2 °C min^−1^) to room temperature.

### Microfluidic chip preparation

The annealed oligonucleotides were transferred to the functionalized glass slides using the micro-contact spotter BioOdyssey Calligrapher® MiniArrayer from Bio-Rad (Hercules, USA) equipped with a solid pin SNS 9 from ArrayIT (Sunnyvale, USA). Each solution was spotted in five replicates with a grid spacing of 1100 μm for the rows and 1300 μm for the columns. The spotting process was carried out at 20 °C and 50% humidity.

During overnight incubation (25 °C, 50% humidity), the oligonucleotides were covalently immobilized via their amino-C12 tags reacting with the terminal epoxy groups of the PEG layer. Remaining binding sites of the surrounding surface were afterwards inactivated with blocking buffer (1 M Tris, 150 mM sodium chloride, pH 8.5, 15 min).

**Fig. 2:**
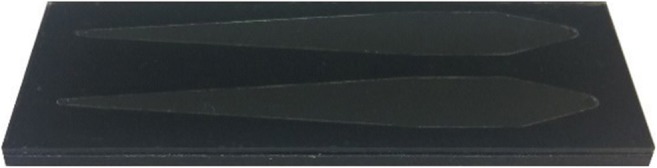
Ready-to-use microfluidic chip (flow direction from left to right)

The flow cell was assembled by connecting the spotted glass slide to a POM sheet (thickness 1 mm) by means of a double-sided adhesive tape ARCare® 90106 from Adhesive Research (Glen Rock, USA) with cutouts forming two flow channels (due to the connections in the MCR 3; only one flow channel was used for this work). Inlet and outlet holes in the POM sheet enabled the passage of fluids. The ready-to-use chips (see Fig. 2) were stored at − 20 °C.

### Measurement setup

The assay was automatically processed by the MCR 3 from GWK Präzisionstechnik (Munich, Germany). The MCR 3 comprises a flow channel for inserting the microfluidic chip, integrated pumps, valves, and tubing for supplying reagents, and a CCD camera for chemiluminescence readout [37]. Assay protocols can be defined and run by the software MCR Visu 1.0.6.2 (GWK Präzisionstechnik, Munich, Germany).

0.5% (w/v) casein in PBS (10 mM potassium dihydrogen phosphate, 70 mM dipotassium hydrogen phosphate, 145 mM sodium chloride) was used as running buffer. The regeneration buffer (100 mM glycine, 100 mM NaCl, and 0.1% (w/v) SDS, adjusted to pH 4) was applied in the last step for removing the bound bio-reagents from the immobilized oligonucleotides.

The biotinylated TetR protein and SA-HRP were diluted in running buffer to a concentration of 0.2 mg L^−1^ and 0.15 mg L^−1^, respectively (stock concentrations 0.6 mg mL^−1^ and 1 mg mL^−1^).

For the chemiluminescence reaction, ready-to-use luminol and hydrogen peroxide solutions from the Elistar Supernova Elisa kit (Cyanagen, Bologna, Italy) were adopted.

All the solutions were filled into the respective reservoirs of the MCR 3 and the tubes were automatically loaded to get ready for measurements.

PBS containing 50 mM MgCl_2_ was used as blank sample for measuring the maximum signal intensities. For calibration experiments, TET was diluted in PBS containing 50 mM MgCl_2_ to defined concentrations (0.01 to 1000 μg L^−1^).

The measurement protocol started with mixing the sample with the TetR solution (1:1) and injecting this mixture into the flow channel of the microfluidic chip. For this step, three different methods were examined: (1) automated mixing by means of the MCR 3 and pumping 400 μL slowly through the flow channel (10 μL s^−1^); (2) stop-flow, pumping each 50 μL of the automated mixed solution slowly in the flow channel (10 μL s^−1^), stop 60 s, repeat the sequence 8 times; (3) manually mixing and injecting 50 μL in the flow channel, incubating for a certain time (10–120 min) and at a defined temperature (18–46 °C) outside the MCR 3.

All the following steps were, again, carried out by means of the MCR 3 at a defined temperature (18 or 37 °C). After cleansing with running buffer (2000 μL with a flow rate of 100 μL s^−1^), the SA-HRP solution was slowly flushed through the flow channel (800 μL with a flow rate of 10 μL s^−1^), followed by another cleansing step (2000 μL with a flow rate of 100 μL s^−1^). A mixture of each 200 μL of hydrogen peroxide and luminol was flushed through the flow channel (100 μL s^−1^). The flow was stopped, and the chemiluminescence was recorded by the CCD camera for 60 s. Subsequently, the whole fluidic system was extensively rinsed with running buffer, and the flow channel was treated with regeneration buffer (1000 μL with a flow rate of 10 μL s^−1^), followed by a final cleansing step (2000 μL with a flow rate of 500 μL s^−1^). Thus, the chip was ready for the next measurement.

The key steps of the established measurement setup are summarized in Table 2.

**Table 2:** Key steps of the established measurement setup

Measurement steps	
1. Manual mixing of sample and TetR solution (each 30 μL)	
2. Injecting 50 μL of the mixture into the microfluidic chip, incubating for a certain time (10–120 min) and at a defined temperature (18–46 °C)	
3. Inserting the chip into the MCR 3 (defined measurement temperature, 18 or 37 °C)	
4. Slowly flushing SA-HRP through the flow channel (800 μL, flow rate 10 μL s^−1^)	
5. Flushing a 1:1 mixture of hydrogen peroxide and luminol through the flow channel (each 200 μL, total flow rate 100 μL s^−1^)	
6. Recording CL with CCD camera for 60 s	
7. Regenerating *tetO* on the chip surface with regeneration buffer (1000 μL, flow rate: 10 μL s^−1^)	

### Data analysis

The detected CL signals were processed with the image evaluation software MCR Image Analyzer 0.3.2.1 (GWK Präzisionstechnik, Munich, Germany).

At the beginning of a measurement day, the background signal of the CCD camera was recorded and then subtracted from each newly recorded CL image. Using the background-corrected CL images, the ten brightest pixels of each spot were averaged. The mean and standard deviation of the five equivalent spots in a row were calculated, excluding spots that deviated more than 20% from the mean. Unspecific CL intensity of the non-spotted chip surface was subtracted from the thus calculated mean values.

To normalize the calibration curves, relative signal intensities *B*/*B*_0_ (in %) were calculated by dividing the signal intensity of a certain TET concentration by the maximum signal intensity obtained in the calibration series (that was set to 100%).

### Analysis of real surface water samples

Nine surface water samples from three sampling sites in the surroundings of a chicken farm in Wullwinkel, Brandenburg, Germany, were collected. After decantation and filtration with syringe filters (pore sizes of 0.8 and 0.22 μm), the samples were directly analyzed.

## Results and discussion

The aim of setting up the assay to gain high and consistent CL signal intensities at the *tetO* spots and low unspecific background. Due to the competitive assay principle, maximum signal intensities were obtained in the absence of TCs. Therefore, the assay characterization was firstly carried out with blank samples and the displayed CL signals were always calculated from the *tetO* signals minus the background intensities.

The decisive factors for the intensities and the reproducibility of the signals were the interaction time for the competition reaction, the temperature during this interaction, the temperature of the following measurement steps, and the DNA oligonucleotide spotting concentration. However, the regenerability was also affected by these parameters, so a systematic investigation was carried out.

### Setup of the competition reaction

In the first step of the assay, the immobilized DNA operator *tetO* and possibly present TC from the sample compete for the receptor protein TetR. For implementing this step, there are three feasible ways to incubate the sample/TetR mixture with immobilized *tetO*: flushing the mixture slowly but constantly through the microfluidic cell, using a stop-flow sequence or a static incubation for an extended time. Compared to a constant flow (10 μL s^−1^) at 18 °C, the stop-flow method with eight increments, each incubated for 1 min, increased the signal intensities by a factor of 2.5, and a static incubation for 5 min or 15 min achieved an increase by a factor of 15 or 25, respectively. While a constant flow had worked for immunoassays [22], the binding of the receptor protein to the double-stranded DNA oligonucleotide obviously required more interaction time.

Thus, the static incubation method was set for further investigations. For practical reasons, the sample/TetR mixture was manually injected in the flow channel of the microfluidic chip and the incubation was performed in an external incubator before the further steps of the assay took place in the MCR 3. Thereby, much less sample and TetR solution was needed per measurement (30 μL instead of 500 μL, respectively), and the MCR 3 could be used for other measurements during the incubation time. In principle, however, fully automated processing of the whole assay in the MCR 3 would be possible as well. Due to requirements of other experiments that were conducted in the same incubator, 37 or 39 °C was used for further experiments.

**Fig. 3:**
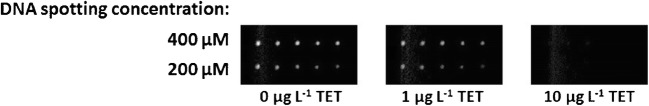
CL images of samples with different TET concentrations. The sample/TetR mixtures were incubated for 30 min at 37 °C, the following measurement steps in the MCR 3 were carried out at 18 °C

It was also demonstrated that a TET content in the sample led to a significant signal decrease (due to the competitive assay principle). Figure 3 shows the CL images of samples with TET concentrations of 0 μg L^−1^, 1 μg L^−1^**,** and 10 μg L^−1^.

### Regeneration of the chip surface

A previous study had shown that the signal intensities of a CL assay with immobilized single-stranded DNA oligonucleotides for the detection of virus and phage DNA/RNA had been significantly increased by performing the entire assay at 40 °C instead of 20 °C [38]. When performing the receptor assay for TC detection at 39 °C instead of 18 °C, the signal intensities were increased by a factor of 4. Since the competition reaction, the binding of streptavidin to biotin and the HRP catalysis are bioreactions as well, they were also more efficient at higher temperature.

**Fig. 4:**
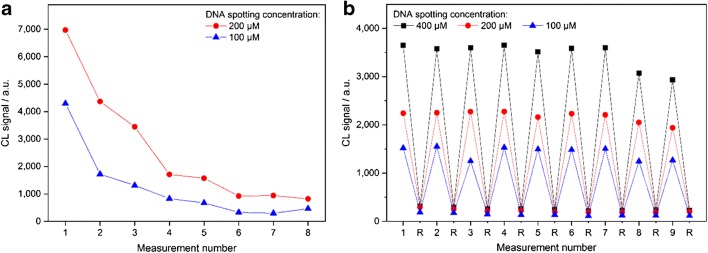
CL signal intensities of consecutive measurements on the same chip. The sample/TetR mixture was each time incubated for 20 min at 39 °C and the following measurement steps in the MCR 3 were carried out at 39 °C (**a**) or 18 °C (**b**), which additionally shows the respective proof-of-regeneration steps (R). As only the trends are relevant, the measurement sequences were conducted once. The points shown in the graphs correspond to the mean values of the five equivalent spots immobilized on the chip in a row, so that signal fluctuations were compensated

An essential feature of this new method, however, should be the regenerability of the double-stranded DNA oligonucleotides immobilized on the chip surface. After recording the signals of the CL reaction, the regeneration was induced by a shift to pH 4 which should change the conformation of the TetR-biotin/SA-HRP complex and thus detach it from the DNA double strand. To evaluate the regenerability of the chip, eight measurements were consecutively carried out at 39 °C (with 20 min incubation of the sample/TetR mixture for each measurement). As shown in Fig. 4a, the resulting CL signal intensities decreased drastically with progressing number of measurements on the same chip. After eight measurements, the remaining signal intensities for DNA spotting concentrations of 200 μM and 100 μM amounted to only 12% and 11% of the first measurement, respectively. The signal loss might be due to the fact that radicals formed from H_2_O_2_ during the CL reaction caused DNA damages based on DNA base oxidation and consecutive break of the double strand [39, 40]. Therefore, the specific DNA operator might have been destructed and no longer been available for the next measurements. Since the radical yield increases with temperature [40], the CL reaction step would be less damaging at lower temperature.

As a promising alternative, the sample/TetR mixture was still incubated at 39 °C whereas the following measurement steps were all conducted at 18 °C. A sequence of nine blank measurements was performed (with 20 min incubation of the sample/TetR mixture for each measurement). After each measurement with regeneration, it was proven in a further step that the surface had been restored to its original state and that there were no remaining signals. For this purpose, the chip was again treated with SA-HRP, followed by luminol and hydrogen peroxide, and another CL image was recorded. Figure 4b shows the resulting CL signals of the sequence with intermediary proof-of-regeneration steps (R).

Compared to the first measurement conducted at 39 °C, the signals amounted to only about one third. However, the signals remained almost constant over the nine measurements. For the DNA spotting concentrations of 400 μM, 200 μM, and 100 μM, the signals obtained in the ninth measurement amounted to 81%, 87%, and 83% of the signals measured at the beginning of the series, respectively. The coefficients of variation between the nine measurements were determined to be 8%, 5%, and 9%, respectively. Thus, the DNA spotting concentration of 200 μM led to the highest regenerability and the lowest signal variations.

The proof-of-regeneration steps (R) showed only very low unspecific signals (less than one tenth of the respective measurement signal), remaining constant over the entire measuring sequence, so the repeated regeneration of the immobilized DNA oligonucleotides was successfully demonstrated.

### Influence of incubation time and temperature

Using the static incubation method, time and temperature of the incubation step were varied to further investigate the influence on the signal intensities.

**Fig. 5:**
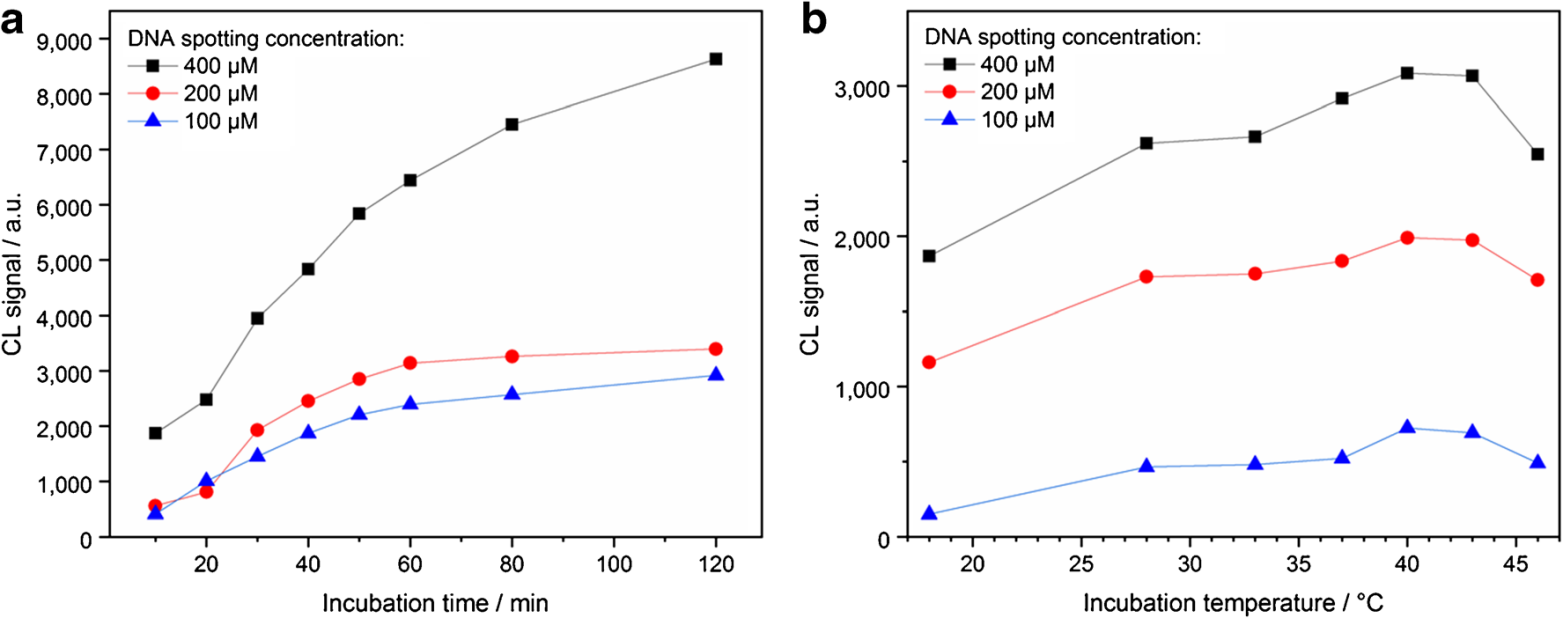
Influence of incubation time (**a**) and temperature (**b**) on the CL signal intensities. The incubation time was varied with a constant temperature of 39 °C (**a**) and the temperature was varied with an incubation time of 15 min (**b**). For both sequences, the following measurement steps in the MCR 3 were carried out at 18 °C. As only the trends are relevant, the measurement sequences were conducted once. The points shown in the graphs correspond to the mean values of the five equivalent spots immobilized on the chip in a row, so that signal fluctuations were compensated

Eight different incubation times between 10 and 120 min were tested (with an incubation temperature of 39 °C and a temperature of 18 °C for the following measurement steps). The resulting CL signal intensities are shown in Fig. 5a. Between 10 and 60 min, there was a strong correlation of incubation time and CL signal intensity. For longer incubation times, the curves became flatter so that a further increase of the incubation time did not have a rewarding effect on the signal intensities. The incubation time for further experiments will always be a trade-off between analysis time and signal yield.

The incubation temperature was varied between 18 and 46 °C in seven steps (with an incubation time of 15 min and a temperature of 18 °C for the following measurement steps). The resulting CL signal intensities are displayed in Fig. 5b. As TetR is a bioreceptor, the curves showed a maximum in the range of 40 °C. At 46 °C, the CL signal intensities decreased significantly due to protein denaturation, so higher temperatures were not examined any more. Performing the competition reaction at biological relevant higher temperatures (37–40 °C) and the further measurement steps at lower temperature (18 °C) turned out to be an efficient concept for regenerable CL receptor assays with immobilized double-stranded DNA oligonucleotides.

### Calibration and recovery experiments

**Fig. 6:**
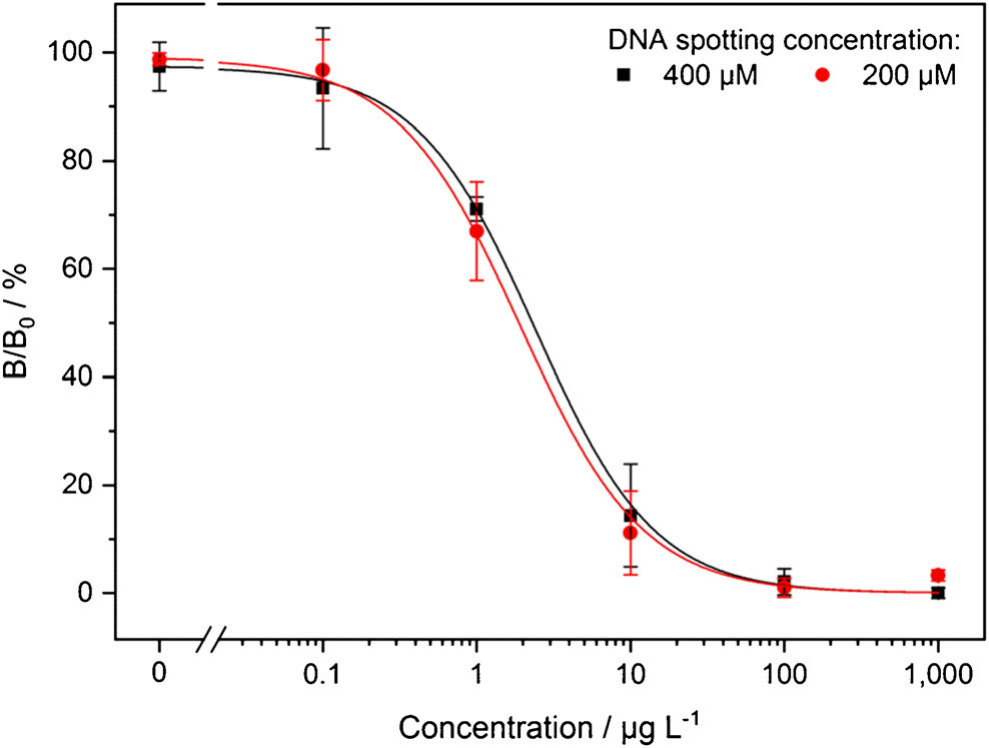
Six-point calibration curves for TET, displayed as relative signal intensities *B*/*B*_0_ (in %) referring to the maximum measured signal of the respective series. Each data point represents the mean of three measurements (carried out on three different chips). The sample/TetR mixture was each time incubated for 30 min at 37 °C, the following measurement steps in the MCR 3 were carried out at 18 °C

To examine the sensitivity and reproducibility of the assay, six-point calibration series with TET concentrations from 0.1 to 1000 μg L^−1^ in tap water were measured on three different chips (with 30 min incubation of the sample/TetR mixture at 37 °C for each measurement). The averaged data points and the sigmoidal regression curves are shown in Fig. 6. For the DNA spotting concentrations of 400 μM and 200 μM, LoDs of 0.5 μg L^−1^ and 0.1 μg L^−1^ and working ranges (80–20% B/B_0_) of 0.6–8.1 μg L^−1^ and 0.5–6.8 μg L^−1^ were obtained, respectively. The lower LoD of 200 μM DNA spotting concentration corresponds with the higher regenerability and the lower coefficient of variation that were determined in the regeneration experiment (see above).

For 1 μg L^−1^ TET spiked in tap water, recoveries of 80% ± 19% and 77% ± 16% for the DNA spotting concentrations of 400 μM and 200 μM, respectively, were obtained.

### Analysis of real surface water samples

The established method is generally applicable for the analysis of real surface water samples as we showed in the introduction that TC concentrations in the μg L^−1^ range were found in regions with intensive livestock farming in Asia and USA. In Europe, however, such high concentrations had never been found so far, so no positive results were expected. Rather, the focus was on investigating whether unspecific signal suppression by the complex real matrix occurs, which would lead to false-positive results.

Nine surface water samples from three sampling sites in the surroundings of a chicken farm in north-eastern Germany were analyzed (with 30 min incubation of the sample/TetR mixture at 37 °C for each measurement). Referring to non-spiked tap water, relative signal intensities of 97% ± 9% for a DNA spotting concentration of 100 μM and 91% ± 8% for a DNA spotting concentration of 200 μM were obtained, meaning that TCs were not detected in any sample. This result showed that signal suppression due to matrix effects did not occur and a pretreatment of the real samples was not required (except for filtration of suspended solids).

## Conclusion and outlook

The presented flow-based regenerable CL receptor assay for the detection of TCs proved to be highly sensitive and regenerable for many runs. The key parameters for high signal yield and regenerability were identified to be (1) the static incubation of the sample/TetR mixture in the flow channel for promoting the competition reaction and (2) the profile of a biologically relevant temperature (37–40 °C) during the competition reaction and a lower temperature (18 °C) for the following measurement steps in order to avoid DNA damage. The incubation time will always be a trade-off between analysis time and signal yield and has therefore to be set concerning the demands of the respective application.

As the measurement protocol was developed based on the already established indirect competitive immunoassays for the multiplex detection of various antibiotics in milk [22] or honey [41] on the microarray analysis platform MCR 3, the concepts can evidently be combined. The future objective is to achieve a single microarray chip that can process multiple receptor and immunoassays in parallel. For competitive immunoassays, the antibiotics of interest can be immobilized besides the DNA oligonucleotides in separate rows on the chip. To perform the assays simultaneously, the corresponding primary antibodies are applied together with TetR (and possibly other receptor proteins) and the HRP-labeled secondary antibody together with streptavidin-HRP. A proof-of-principle study combined the TC receptor assay with some indirect competitive immunoassays. The measurements were performed with non-spiked tap water samples in the same way as it was optimized for only TC. When TetR and the primary antibodies were mixed, all the different spotted rows gave signals. When only TetR or the primary antibodies were applied, only the *tetO* row or the respective antibiotic rows appeared. Thus, it was proven that there were no cross reactivities and that the combination is basically possible. A detailed study with calibrating all the assays, however, is very complex and effortful, so this will be the subject of further research.

In a future perspective, the monitoring of surface and sewage waters will become a major topic in environmental analysis [42, 43]. Quality requirements have already been specified, e.g., in the European Water Framework Directive (WFD) 2000/60/EC. An unsolved problem is that wastewater treatment plants are not yet able to completely remove micropollutants such as antibiotics from wastewater [44], so these compounds are drained in the environment. Furthermore, heavy rainfall events, which are increasing as a result of climate change [45, 46], mobilize micropollutants adsorbed to the soil and therefore flush them into surface waters [47]. In order not only to carry out isolated analyses, but also to ensure continuous monitoring, an online measuring device is required.

It has already been shown that the TC sensor works for real surface waters without pretreatment, although no TCs have been detected so far in a single sampling campaign in Germany. Profound studies in other countries will underline the efficiency of this innovative method. Both the multiplexing ability and the reusability make the sensor ideally suited for a fully automated monitoring of multiple contaminants in the field.

## Abbreviations

CCD: Charge-coupled device
CL: Chemiluminescence
CTC: Chlortetracycline
DNA: Deoxyribonucleic acid
DOX: Doxycycline
ELISA: Enzyme-linked immunosorbent assay
His_6_/His_5_: Hexa/pentahistidine
HPLC-MS: High-performance liquid chromatography–mass spectrometry
HRP: Horseradish peroxidase
IgG: Immunoglobulin G
LoD: Limit of detection
MCR 3: Microarray Chip Reader (3rd generation)
MIPs: Molecularly imprinted polymers
MTP: Microtiter plate
OTC: Oxytetracycline
PEG: Polyethylene glycol
POM: Polyoxymethylene
SA-HRP: HRP-conjugated streptavidin
SDS: Sodium dodecyl sulfate
SPR: Surface plasmon resonance
TC: Tetracycline derivative
TET: Tetracycline
*tetO*: Tetracycline operator DNA sequence
TetR: Tetracycline repressor protein
TetA: Tetracycline efflux protein
Tris: Tris(hydroxymethyl)aminomethane
